# A Three‐Way Comparison of Nodular Lesions in *H. pylori*‐Induced Gastritis, Non‐*Helicobacter pylori Helicobacter* (NHPH)‐Induced Gastritis, and NHPH‐Induced MALT Lymphoma Reveals Their Distinct Endoscopic Predictors: Distribution for Bacterial Etiology and Morphology for Malignancy

**DOI:** 10.1111/hel.70079

**Published:** 2025-10-14

**Authors:** Yuki Kitadai, Hidehiko Takigawa, Akinori Nagao, Daisuke Shimizu, Misa Ariyoshi, Takeshi Takasago, Akiyoshi Tsuboi, Hidenori Tanaka, Ken Yamashita, Yuichi Hiyama, Yoshihiro Kishida, Yuji Urabe, Akira Ishikawa, Toshio Kuwai, Shiro Oka

**Affiliations:** ^1^ Department of Gastroenterology, Graduate School of Biomedical and Health Sciences Hiroshima University Hiroshima Japan; ^2^ Department of Molecular Pathology, Graduate School of Biomedical and Health Sciences Hiroshima University Hiroshima Japan; ^3^ Gastrointestinal Endoscopy and Medicine Hiroshima University Hospital Hiroshima Japan

**Keywords:** *Helicobacter pylori*, mucosa‐associated lymphoid tissue lymphoma, NHPH, nodular gastritis, non‐*Helicobacter pylori Helicobacter*

## Abstract

**Background:**

Nodular gastritis associated with *Helicobacter pylori* infection (HPi‐NG) has long been recognized. Recently, similar mucosal changes have been reported with non‐*H. pylori Helicobacter* (NHPH), including NHPH‐induced gastric mucosa‐associated lymphoid tissue lymphoma with a nodular gastritis‐like appearance (NHPHi‐MNG) and NHPH‐induced nodular gastritis (NHPHi‐NG). However, how bacterial species differences and the presence or absence of malignancy are reflected in endoscopic appearance has not been sufficiently investigated. This study aimed to clarify these relationships and to identify key endoscopic features that differentiate among the three clinically distinct entities.

**Methods:**

We retrospectively analyzed 69 patients diagnosed at Hiroshima University Hospital between 2013 and 2024: 11 with NHPHi‐MNG, 12 with NHPHi‐NG, and 46 with HPi‐NG. Endoscopic findings were compared, focusing on three morphological features of the nodules (maximum diameter, size heterogeneity, and shape) and three distributional features (extent of nodularity, extension to the lower gastric body, and predominant site of nodularity), along with patient sex. Diagnostic accuracy was calculated based on combinations of these features.

**Results:**

NHPHi‐MNG was differentiated from NHPHi‐NG with 91.3% accuracy when two or more of the three morphological criteria were satisfied. NHPHi‐MNG was distinguished from HPi‐NG with 93.0% accuracy when four or more of seven criteria were fulfilled: the three morphological features, three distributional features, and patient sex. NHPHi‐NG was differentiated from HPi‐NG with 91.4% accuracy when all three distributional features were present.

**Conclusion:**

Among the three nodular gastric conditions, the morphological features of nodules reflected the presence or absence of malignancy, whereas the distributional features were associated with the underlying bacterial species. These findings suggest that specific endoscopic features can aid in distinguishing between benign and malignant forms of nodular gastric lesions and between infections with different *Helicobacter* species, providing important diagnostic guidance.

## 1 Introduction

Nodular gastritis has traditionally been recognized as a distinct form of gastritis associated with *Helicobacter pylori* (HP) infection. It is endoscopically characterized by diffusely distributed nodular lesions, primarily observed in the antrum of the stomach [1, 2, 3]. In recent years, endoscopic nodularity has also been reported in non‐HP *Helicobacter* (NHPH) infection, occurring in nodular gastritis as well as in cases diagnosed with gastric mucosa‐associated lymphoid tissue (MALT) lymphoma, highlighting their increasing clinical relevance [4, 5, 6]. In particular, three disease entities—NHPH‐induced gastric MALT lymphoma with a nodular gastritis‐like appearance (NHPHi‐MNG), NHPH‐induced nodular gastritis (NHPHi‐NG), and HP‐induced nodular gastritis (HPi‐NG)—often present with remarkably similar endoscopic features, making differentiation difficult in clinical practice. However, these three conditions represent distinct pathological entities with different bacterial species and malignant or benign statuses. Accurate diagnosis is essential, as it considerably influences subsequent diagnostic evaluations and therapeutic decisions. We previously reported that endoscopic differences in the morphology and distribution of nodules could help distinguish NHPHi‐MNG from HPi‐NG [7]. Nonetheless, it remains unclear whether these differences reflect underlying malignancy or simply differences in bacterial species, and this distinction has not been rigorously examined. Although several studies have investigated endoscopic differences between HP‐induced and NHPH‐induced gastritis [8, 9], no study to date has specifically focused on nodular‐type gastritis.

Although nodular gastritis has classically been linked to HP [1, 2], widespread eradication has reduced HP prevalence [10, 11], ushering in a post‐eradication era in which NHPH‐associated diseases are increasingly encountered in routine practice. Once considered extremely rare, NHPH infection is now recognized—through advances in polymerase chain reaction (PCR) and immunohistochemistry—to be more common than expected in HP‐negative gastritis and gastric MALT lymphoma [8, 12, 13]. Nakamura et al. reported that approximately 21% of HP‐negative patients were NHPH‐positive, with *H. suis* being the most frequently identified species.

Whereas HP has well‐established diagnostic methods in routine use, NHPH lacks standardized testing and currently requires direct detection in biopsy specimens or PCR‐based identification [14]. However, PCR is available only in limited facilities, and NHPH is known to be unevenly distributed within the gastric mucosa, increasing the risk of false negatives depending on the biopsy site [15, 16]. Furthermore, distinguishing NHPH from HP based solely on histological morphology is often difficult, presenting ongoing challenges in NHPH diagnosis. Although several innovative approaches—such as enzyme‐linked immunosorbent assay (ELISA)‐based [17] and culture‐based [18] methods—have recently been reported, they are not yet ready for clinical application.

Gastric MALT lymphoma is a low‐grade B‐cell lymphoma derived from MALT, typically induced by chronic inflammation [19, 20]; early lesions can mimic reactive lymphoid follicles. Histopathological diagnosis is based on findings such as diffuse infiltration of atypical B cells, lymphoepithelial lesions (LELs), and evidence of monoclonality by immunoglobulin heavy chain (IgH) gene rearrangement. However, in borderline lesions, where these findings are unclear, distinguishing between neoplastic and non‐neoplastic processes can be challenging. In such cases, diagnosis cannot rely on histological findings alone, and a comprehensive evaluation, including the clinical course, endoscopic findings, and response to eradication therapy, is necessary.

Given this background, accurate differentiation and diagnosis of the three conditions—HPi‐NG, NHPHi‐NG, and NHPHi‐MNG—has become a challenging yet critical issue in clinical settings during the post‐eradication era. Furthermore, to the best of our knowledge, no comprehensive cross‐sectional analysis has directly compared all three entities—HPi‐NG, NHPHi‐NG, and NHPHi‐MNG—in the context of nodular endoscopic appearance. Therefore, in this study, we aimed to clarify how differences in bacterial species and malignancy are reflected in the endoscopic features of nodular gastritis‐like lesions.

In this study, we conducted a cross‐sectional comparison of nodular findings among the three diseases to clarify the differences in the morphological and distributional characteristics of the nodules. Furthermore, we aimed to identify endoscopic differentiation points by considering the underlying pathophysiological mechanisms that may contribute to these differences.

## 2 Methods

### 2.1 Patients

**FIGURE 1:**
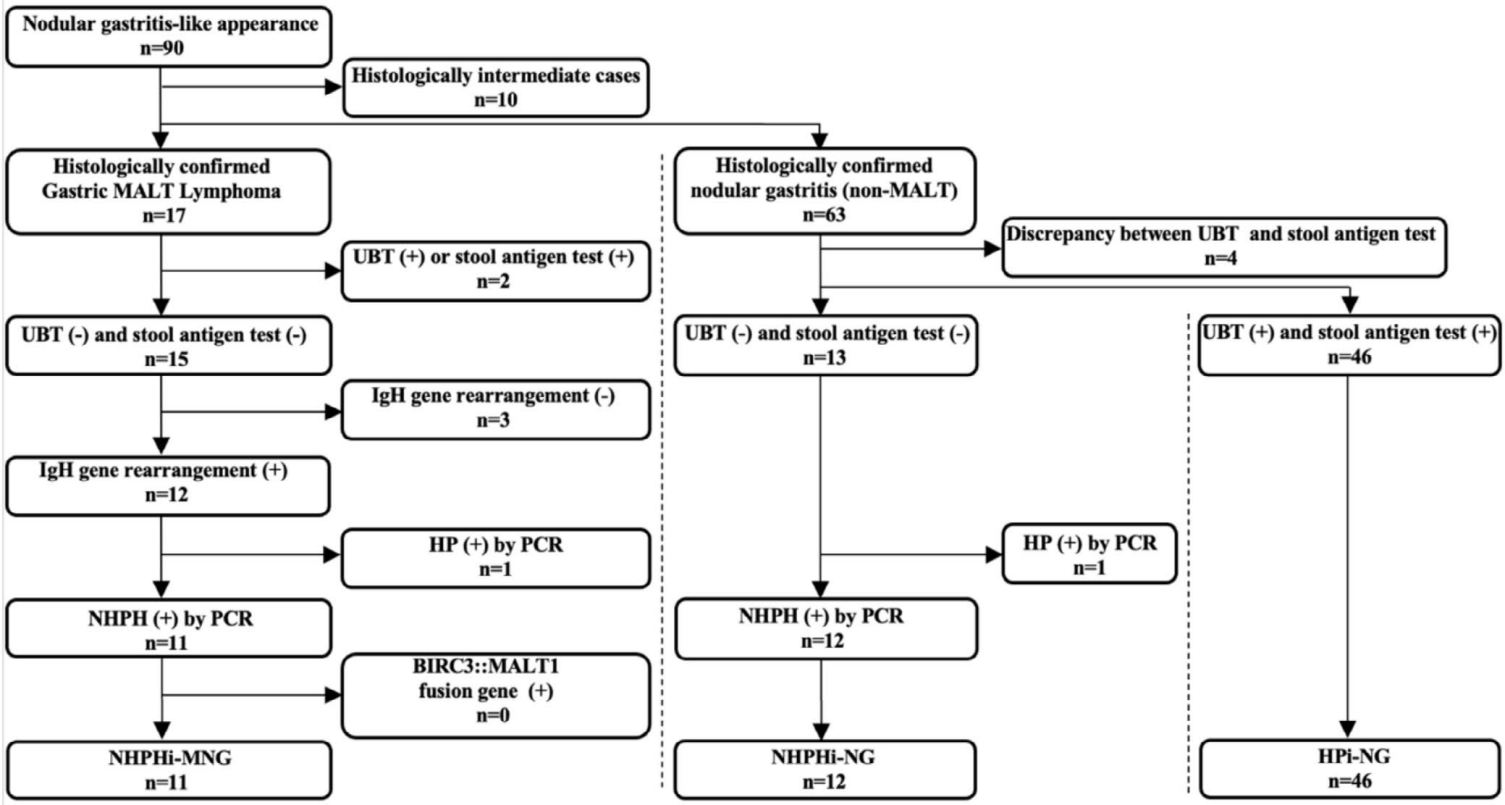
Flowchart of patient selection for NHPHi‐MNG, NHPHi‐NG, and HPi‐NG. This flowchart outlines the selection of patients who underwent EGD at Hiroshima University Hospital between 2013 and 2024 with a nodular gastritis‐like appearance. Of 90 eligible cases, 10 histologically intermediate cases were excluded. The remaining 80 cases included 17 patients histologically diagnosed with gastric MALT lymphoma and 63 with benign nodular gastritis. Among the MALT lymphoma cases, 11 were classified as NHPHi‐MNG based on the following criteria: negative UBT and stool antigen test, positive IgH gene rearrangement, PCR‐confirmed HP negativity and NHPH positivity, and absence of BIRC3::MALT1 fusion gene. Among the 63 benign nodular gastritis cases, four with discrepant UBT and stool antigen test results were excluded. Of the remaining 59 cases, 12 with negative UBT and stool antigen tests, PCR‐negative for HP, and PCR‐positive for NHPH were classified as NHPHi‐NG. The 46 cases with both UBT and stool antigen test positivity were categorized as HPi‐NG. These three groups were analyzed comparatively in terms of clinical, pathological, and endoscopic features. EGD, esophagogastroduodenoscopy; HP, *Helicobacter pylori* ; HPi‐NG, *Helicobacter pylori* ‐induced nodular gastritis; IgH, immunoglobulin heavy chain; NHPH, non‐*Helicobacter pylori Helicobacter*; NHPHi‐MNG, non‐*Helicobacter pylori Helicobacter*‐induced gastric mucosa‐associated lymphoid tissue lymphoma with a nodular gastritis‐like appearance; UBT, urea breath test.

We retrospectively reviewed 90 patients who underwent esophagogastroduodenoscopy (EGD) at Hiroshima University Hospital between 2013 and 2024 and exhibited a nodular gastritis‐like appearance. Among these, 10 cases with borderline lesions, in which pathological assessment could not definitively distinguish between neoplastic and non‐neoplastic changes, were excluded. The remaining patients were classified into two groups: 17 cases diagnosed with gastric MALT lymphoma and 63 cases with benign nodular gastritis showing no evidence of neoplastic transformation. Of the 17 MALT lymphoma cases, 11 tested negative for both the urea breath test (UBT) and stool HP antigen, showed positive IgH gene rearrangement, and were confirmed to be HP‐negative and NHPH‐positive by PCR. These cases were defined as NHPHi‐MNG. All NHPHi‐MNG cases were negative for the BIRC3::MALT1 fusion gene. Among the 63 cases of benign nodular gastritis, four were excluded because of discordant results between UBT and stool antigen testing. Of the remaining 59 cases, 12 were negative for both UBT and stool HP antigen and confirmed to be HP‐negative and NHPH‐positive by PCR; these were defined as NHPHi‐NG. The other 46 cases, which were positive for both UBT and stool HP antigen, were classified as HPi‐NG. The clinical, pathological, and endoscopic characteristics of the three groups were compared (Figure 1). Given the potential effects on endoscopic and inflammatory findings [21, 22, 23, 24, 25], we confirmed that no participants were taking nonsteroidal anti‐inflammatory drugs, proton‐pump inhibitors, potassium‐competitive acid blockers, or H2‐receptor antagonists at the time of EGD in the final analytic cohort.

### 2.2 Differentiation Between Nodular Gastritis and Gastric MALT Lymphoma

The pathological diagnosis of gastric MALT lymphoma was based on the World Health Organization Classification of Tumors, 5th edition [26], and was conducted by an experienced gastrointestinal pathologist specializing in malignant lymphomas. Diagnostic criteria included the presence of lymphoepithelial lesion (LEL), follicular colonization by tumor cells, and architectural abnormalities such as follicular fusion and loss of polarity. LELs were identified using hematoxylin and eosin (H&E) staining and further evaluated by immunohistochemistry using cytokeratin AE1/AE3 and CD20 when necessary. Additional markers, including CD10, Bcl‐2, and Ki‐67, were used to assess changes in the follicular architecture and proliferation patterns. As LEL‐like features can occasionally be observed in benign nodular gastritis, the presence of LEL alone is not a definitive diagnostic marker. Instead, emphasis was placed on the combination of architectural abnormalities and infiltrative patterns suggestive of malignancy. Cases classified as borderline lesions, in which it was difficult to definitively determine neoplastic status, were excluded from the present study.

To confirm monoclonality, a key diagnostic criterion for MALT lymphoma, IgH gene rearrangement analysis was performed using the IGH Clonality Assay (Invivoscribe Technologies, San Diego, CA, USA) following the manufacturer's instructions [27]. Although IgH gene rearrangement has a reported positivity rate of 48%–91% in MALT lymphoma [28, 29], false positives have been observed in approximately 10% of gastritis cases [30]. Therefore, IgH positivity alone was not considered sufficient for diagnosis and was interpreted in conjunction with other pathological findings. The BIRC3::MALT1 fusion gene was detected using fresh biopsy specimens collected from all patients. Fluorescence in situ hybridization analysis was conducted at LSI Medience Corporation (Tokyo, Japan) following previously established methods [31, 32].

### 2.3 Evaluation of *Helicobacter* Infection Status: HP vs. NHPH

To determine the infecting *Helicobacter* species, all patients underwent both a UBT and a stool antigen test. HP infection was defined strictly as cases testing positive on both UBT and stool antigen testing to minimize the possibility of co‐infection with NHPH. This definition was based on previous reports indicating that although UBT may yield positive results in NHPH infection, stool antigen testing demonstrates high specificity for HP, with all reported NHPH cases testing negative by this method [33].

Conversely, NHPH infection was defined as cases negative on both UBT and stool antigen testing. To confirm the infecting species, DNA was extracted from gastric biopsy specimens collected from the antrum, angulus, and corpus. PCR assays specific for HP and NHPH were then performed. DNA extraction was performed using the AllPrep DNA/RNA Kit (Qiagen Japan, Tokyo, Japan), and DNA concentrations were measured using a NanoDrop spectrophotometer. PCR was conducted using the KOD FX Neo kit (TOYOBO, Osaka), including its buffer system and DNA polymerase, to amplify the urease gene regions specific to NHPH species, including *H. suis* , *H. bizzozeronii* , *H. felis* , *H. salomonis* , and *H. heilmannii s.s*. The PCR conditions and primers were used as described in a previous study [34].

### 2.4 Characterization of Nodular Gastric Findings on Endoscopy

Endoscopic images with minimal distortion and adequate observational conditions obtained from EGD were retrospectively reviewed by three board‐certified gastrointestinal endoscopists (> 10 years' experience), all members of our institutional MALT/NHPH team with expertise in NHPH‐related diseases and gastric MALT lymphoma. Reviewers were blinded to the final diagnosis and to each other's measurements, and image interpretation was performed based on their standard clinical judgment.

Indigo carmine improves visualization of subtle nodules—clarifying borders, surface irregularities, and extent—and thereby supports targeted biopsy and differentiation between nodular gastritis and gastric MALT lymphoma [4, 35, 36, 37, 38, 39]. We, therefore, evaluated all nodular lesions using indigo carmine chromoendoscopy. The evaluation focused on the distribution and morphological features of nodular lesions. For distributional analysis, the extent of nodularity, distance from the angulus to the proximal margin of the nodules, and predominant location of nodularity were assessed. Regarding morphology assessment, evaluations were based on the maximum diameter of nodules, the degree of size heterogeneity, and nodule shape. Nodules were classified as either protruded type (marked elevation) or flat type (minimal elevation). For categorical variables, the final call was based on agreement by at least two of the three reviewers. For continuous variables (distance from the gastric angulus to the proximal end of nodular lesion and maximum nodule diameter), measurements were recorded to the nearest 0.5 cm and 1 mm, respectively, and the median of the three measurements was used for analysis. The diagnostic utility of these endoscopic features was then analyzed to determine their effectiveness in differentiating among the three disease entities—NHPHi‐MNG, NHPHi‐NG, and HPi‐NG. Additionally, to provide a bedside decision aid, we trained a classification and regression tree (CART) using the rpart package in R, based on endoscopic features significant on univariable analyses. Continuous variables were dichotomized using exploratory, data‐driven cutoffs.

### 2.5 Pathology–Endoscopy Correlation

To assess whether endoscopic nodules correspond to lymphoid follicles and to justify using maximum nodule diameter as the primary endpoint, we correlated the endoscopic maximum nodule diameter with the maximum follicle diameter on H&E‐stained sections. For cases with multiple biopsies, the largest follicle and the largest nodule per case were used (one datum per case); coalescent follicles were treated as a single mass. A total of 26 biopsy‐proven follicle–positive cases were analyzed.

### 2.6 Statistical Analysis

All statistical analyses were performed using EZR (Saitama Medical Center, Jichi Medical University, Saitama, Japan). For continuous variables, the Mann–Whitney *U* test or Student's *t*‐test was used, depending on the data distribution. For categorical variables, the chi‐square (*χ* ^2^) test or Fisher's exact test was applied as appropriate. All statistical tests were two‐sided, and *p* values < 0.05 were considered significant. For multiple group comparisons, one‐way analysis of variance followed by Bonferroni post hoc correction was used [40]. Bivariate correlation between endoscopic and pathological measurements was assessed using Pearson's correlation coefficient (two‐sided). For analyses involving ordinal variables or non‐normally distributed data, Spearman's rank correlation was applied, and ρ values with two‐sided *p*‐values were reported.

Interobserver agreement for continuous measurements was assessed using ICC(2,1)—two‐way random effects, absolute agreement, single measurement—calculated with the irr package in R (icc(model = “twoway”, type = “agreement”, unit = “single”)) [41, 42]. Ninety‐five percent confidence intervals were derived from the F distribution. Interpretation followed established thresholds: poor < 0.50, moderate 0.50–0.75, good 0.75–0.90, excellent > 0.90 [42]. ICCs were computed from the three individual rater measurements per case, prior to aggregation.

Following prior reports [43], we trained a CART model using rpart (R) [44, 45]. Data were stratified 80/20 into a training set (*n* = 56) and an independent test set (*n* = 13). Candidate endoscopic predictors were selected based on significance in the training set univariable analyses, and continuous predictors were dichotomized at exploratory cutoffs. In the training set, a maximal tree (cp = 0) was grown and pruned using the 1‐SE rule with stratified 10‐fold cross‐validation. All predictors were entered as categorical variables. Performance was estimated from out‐of‐fold predictions using overall accuracy, Cohen's *κ*, macro‐averaged balanced accuracy, and class‐wise sensitivity, specificity, and positive predictive value (PPV), and subsequently evaluated on the test set using the same metrics (accuracy with 95% CI).

## 3 Results

### 3.1 Patient Characteristics

The clinical characteristics of the enrolled patients are summarized in Table 1. The study population was classified into three groups: NHPHi‐MNG (*n* = 11), NHPHi‐NG (*n* = 12), and HPi‐NG (*n* = 46). The median age (range) was 52 years (39–76) in the NHPHi‐MNG group, 39 years (13–60) in the NHPHi‐NG group, and 41 years (12–76) in the HPi‐NG group. There was no significant difference in age among the three groups (*p* = 0.090). Regarding sex distribution, the proportion of male patients was significantly higher in the NHPHi‐MNG group (73%, 8/11) than in the HPi‐NG group (24%, 11/46) (*p* = 0.011). No significant differences were observed between the NHPHi‐MNG and NHPHi‐NG or between the NHPHi‐NG and HPi‐NG groups. With respect to mucosal atrophy (Kimura–Takemoto classification), all cases in both the NHPHi‐MNG and NHPHi‐NG groups were none/closed‐type (100% each). In the HPi‐NG group, 80% (37/46) were none/closed‐type, and 20% (9/46) were open‐type; this difference did not reach statistical significance (*p* = 0.102). Regarding eradication history, all patients in both the NHPHi‐MNG and NHPHi‐NG groups had undergone eradication therapy only once, whereas 13% (6/46) of patients in the HPi‐NG group had received two courses; however, this difference was not significant (*p* = 0.292). Following eradication therapy, nodular lesions completely disappeared in all cases in both the NHPHi‐MNG and NHPHi‐NG groups. Similarly, 93% (43/46) of HPi‐NG cases showed complete resolution of nodules (*p* = 1.000). The three cases with persistent nodules were all resistant to eradication therapy. The BIRC3::MALT1 fusion gene test was performed only in cases pathologically diagnosed as MALT lymphoma, and all tested cases were negative.

**TABLE 1:** Comparison of patient characteristics and endoscopic findings among the NHPHi‐MNG, NHPHi‐NG, and HPi‐NG groups.

	a. NHPHi‐MNG, *n* = 11	b. NHPHi‐NG, *n* = 12	c. HPi‐NG, *n* = 46	*p* ^a^ (Total)	*p* ^b^ (a vs. b)	*p* ^b^ (a vs. c)	*p* ^b^ (b vs. c)
Age [range]	52 [39–76]	39 [13–60]	41 [12–76]	0.090			
*Sex*							
Female	3 (27)	6 (50)	35 (76)	0.006*	1.000	0.011*	0.450
Male	8 (73)	6 (50)	11 (24)				
*Mucosal atrophy*							
None, Closed‐type	11 (100)	12 (100)	37 (80)	0.102	—	—	—
Open‐type	0 (0)	0 (0)	9 (20)				
*Number of HP eradication treatments*							
Once	11 (100)	12 (100)	40 (85)	0.292	—	—	—
Twice	0 (0)	0 (0)	6 (15)				
*Disappearance of nodules*							
Yes	11 (100)	12 (100)	43 (93)	1.000	—	—	—
*After eradication*							
No	0 (0)	0 (0)	3 (7)	1.000	—	—	—
*BIRC3::MALT1 fusion gene*							
Positive	0 (0)	0 (0)	—	—	—	—	—
Negative	11 (100)	12 (100)	—				
*Extent of nodularity*							
Antrum alone	0 (0)	0 (0)	20 (43)	0.003*	1.000	0.016*	0.014*
Beyond the angulus	11 (100)	12 (100)	26 (57)				
*Predominant site of nodularity*							
Antrum	3 (27)	4 (33)	45 (98)	< 0.001*	1.000	< 0.001*	< 0.001*
Angulus	8 (73)	8 (67)	1 (2)				
Distance from gastric angulus to the proximal end of nodular lesion ± SE (cm)	3.5 ± 1.0	3.1 ± 0.7	1.7 ± 1.6	< 0.001*	1.000	< 0.001*	0.007*
Maximum diameter of nodules ± SE (mm)	3.3 ± 0.7	2.3 ± 0.5	2.1 ± 0.4	< 0.001*	< 0.001*	< 0.001*	0.43
*Size heterogeneity of nodules*							
Uniform	4 (36)	12 (100)	42 (91)	< 0.001*	0.004*	< 0.001*	1.000
Heterogeneous	7 (64)	0 (0)	4 (9)				
*Shape of nodules*							
Flat type	3 (27)	12 (100)	40 (87)	< 0.001*	0.001*	< 0.001*	0.98
Protruded type	8 (73)	0 (0)	6 (13)				
	(%)	(%)	(%)				

*Note:* Post hoc analysis was performed with Bonferroni correction (*p* < 0.05/3). *p < 0.05, **p < 0.05/3.

Abbreviations: HP, *Helicobacter pylori* ; HPi‐NG, *Helicobacter pylori* ‐induced nodular gastritis; NHPH, non‐*Helicobacter pylori Helicobacter*; NHPHi‐MNG, non‐*Helicobacter pylori Helicobacter*‐induced gastric mucosa‐associated lymphoid tissue lymphoma with a nodular gastritis‐like appearance; SE, standard error.

^a^ One‐way ANOVA was used for continuous variables, and a 2 × 3 Fisher's exact test was used for categorical variables. Statistical significance was set at *p* < 0.05.

^b^ When the *p* value in the one‐way analysis among the three groups was significant, pairwise comparisons were performed using Student's *t*‐test for continuous variables and Fisher's exact test for categorical variables.

### 3.2 Comparison of Endoscopic Features of Nodular Lesions

**FIGURE 2:**
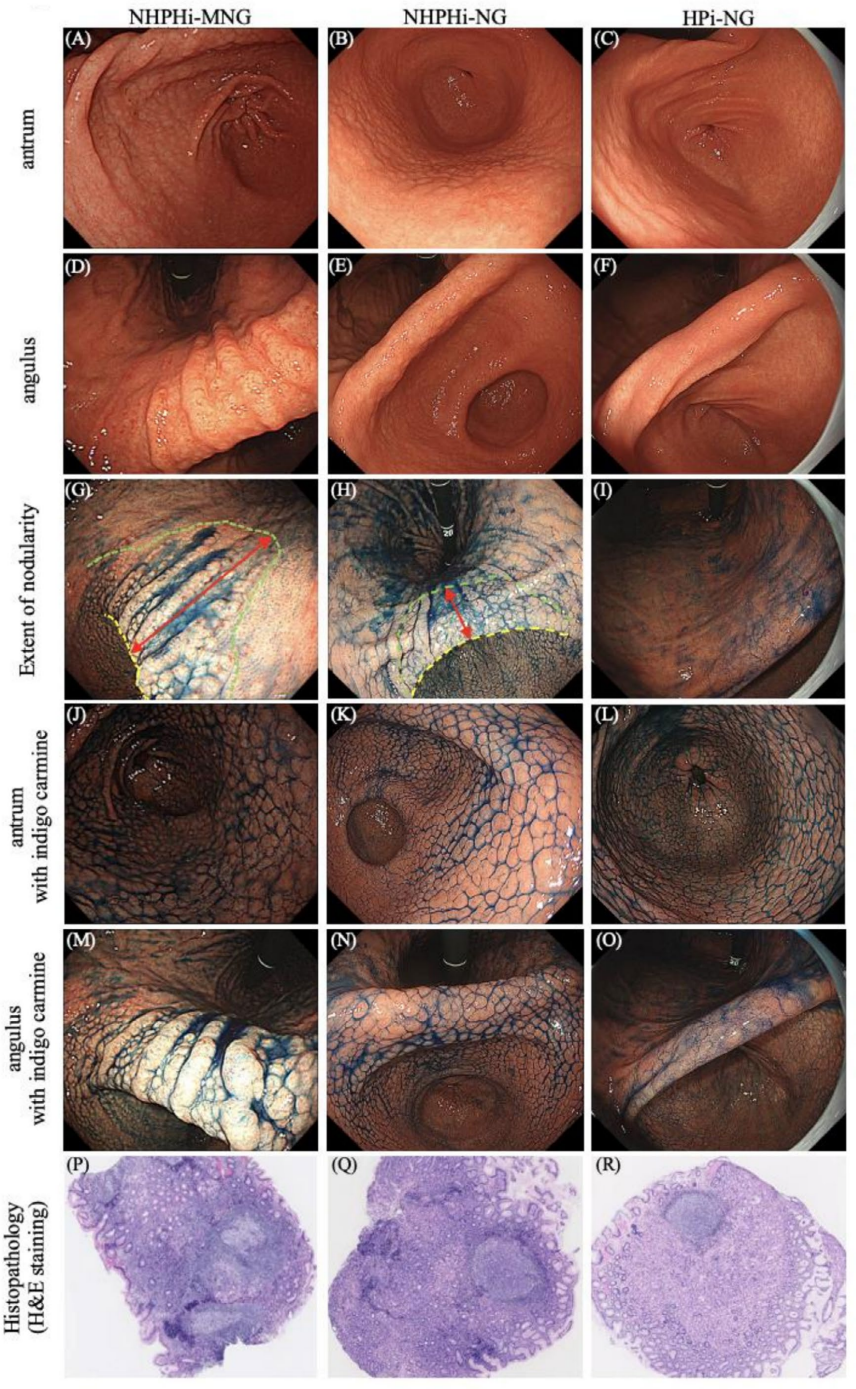
Comparative endoscopic and pathological findings among NHPHi‐MNG, NHPHi‐NG, and HPi‐NG. Conventional white‐light endoscopic images of the antrum and angulus. Nodules in the NHPHi‐MNG group appear larger, more heterogeneous in size, and more elevated compared to the relatively flat, uniform nodules observed in both the NHPHi‐NG and HPi‐NG groups (A–F). Method for measuring the distance from the gastric angulus to the proximal end of nodular lesion. The yellow and green dashed lines indicate the gastric angulus and the proximal end of nodular lesion, respectively; the red arrow shows the measured distance between them. Nodules in the NHPHi‐MNG and NHPHi‐NG groups frequently extend beyond the angulus into the lower corpus, while those in the HPi‐NG group often remain confined to the antrum (G–I). Indigo carmine chromoendoscopy of the antrum and angulus. Nodular patterns are more accentuated with indigo carmine. In NHPHi‐MNG and NHPHi‐NG, nodular changes are most pronounced in the angulus, whereas in HPi‐NG, they are most intense in the antrum (J–O). Representative histological findings. In the NHPHi‐MNG group, prominent lymphocytic infiltration with irregular, fused follicle‐like structures lacking polarity and size uniformity was observed. In contrast, both the NHPHi‐NG and HPi‐NG groups exhibited well‐polarized, uniform lymphoid follicles (P–R). H&E, hematoxylin and eosin; HP, *Helicobacter pylori* ; HPi‐NG, HP‐induced nodular gastritis; NHPH, non‐*Helicobacter pylori Helicobacter*; NHPHi‐MNG, NHPH‐induced gastric mucosa‐associated lymphoid tissue lymphoma with a nodular gastritis‐like appearance; NHPHi‐NG, NHPH‐induced nodular gastritis.

**FIGURE 3:**
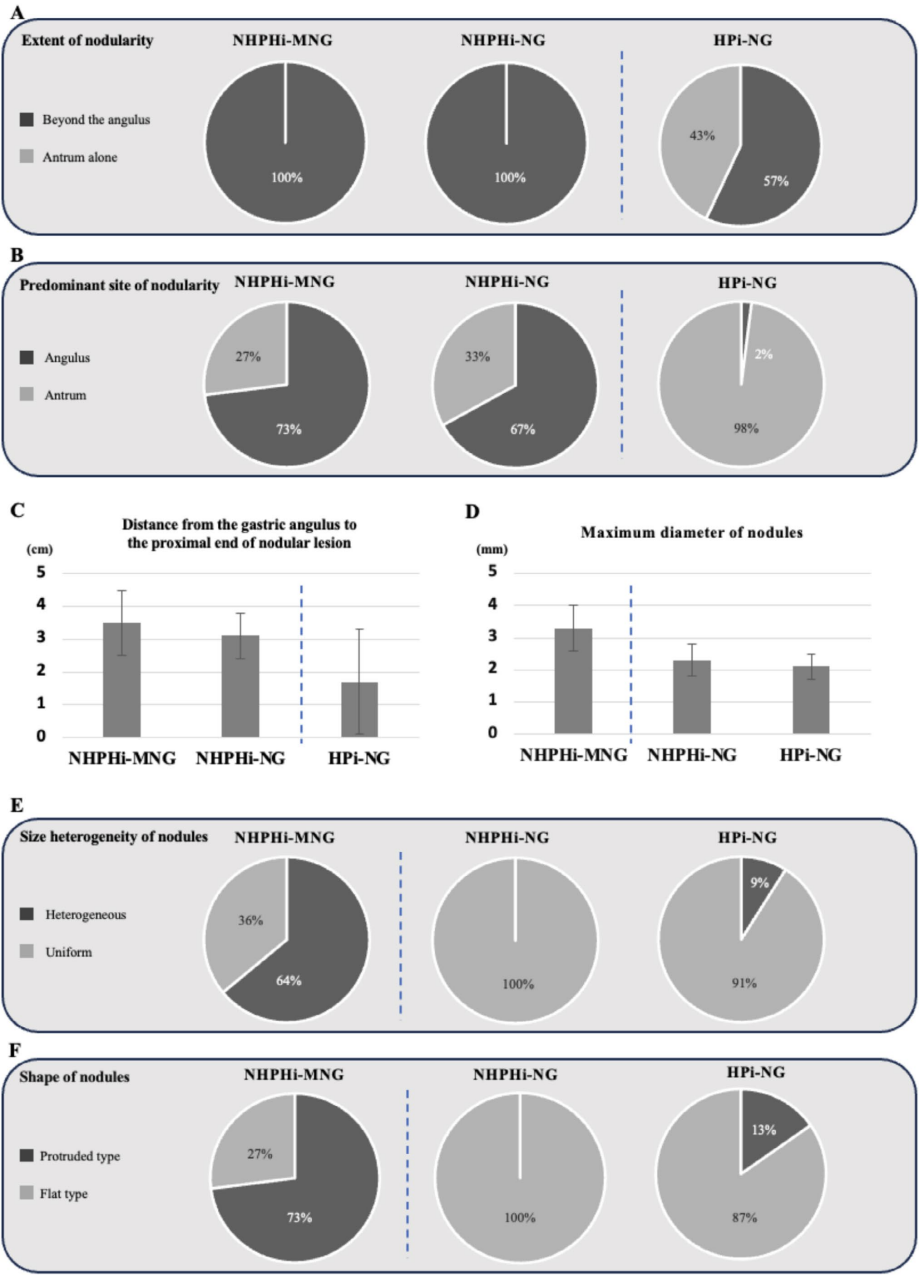
Comparison of endoscopic features among NHPHi‐MNG, NHPHi‐NG, and HPi‐NG. (A) Extent of nodularity: All cases in the NHPHi‐MNG and NHPHi‐NG groups exhibited lesions extending beyond the gastric angulus, while in the HPi‐NG group, lesions were limited to the antrum in 57% of cases. (B) Predominant site of nodularity: In the NHPHi‐MNG and NHPHi‐NG groups, nodular changes were most frequently predominant in the angulus (73% and 67%, respectively), whereas in the HPi‐NG group, 98% of cases showed the most prominent nodularity in the antrum. (C) Distance from the gastric angulus to the proximal end of nodular lesion: Both the NHPHi‐MNG and NHPHi‐NG groups showed a significantly longer distance than the HPi‐NG group. (D) Maximum diameter of nodules: Nodules in the NHPHi‐MNG group were larger on average than those in the NHPHi‐NG and HPi‐NG groups. (E) Size heterogeneity of nodules: The NHPHi‐MNG group showed greater heterogeneity (36%) than the nearly uniform distribution observed in the NHPHi‐NG (100%) and HPi‐NG (91%) groups. (F) Shape of nodules: A higher proportion of protruded‐type nodules was observed in the NHPHi‐MNG group (27%) than the 0% and 13% observed in the NHPHi‐NG and HPi‐NG groups, respectively. HP, *Helicobacter pylori* ; HPi‐NG, HP‐induced nodular gastritis; NHPH, non‐*Helicobacter pylori Helicobacter*; NHPHi‐MNG, NHPH‐induced gastric mucosa‐associated lymphoid tissue lymphoma with a nodular gastritis‐like appearance; NHPHi‐NG, NHPH‐induced nodular gastritis.

Regarding lesion distribution (Figure 2A–F), all cases in the NHPHi‐MNG and NHPHi‐NG groups showed nodular extension beyond the gastric angulus into the corpus, whereas 43% (20/46) of HPi‐NG cases were confined to the antrum (Figure 3A). This difference was significant (NHPHi‐MNG vs. HPi‐NG: *p* = 0.016; NHPHi‐NG vs. HPi‐NG: *p* = 0.014). The site of most prominent nodular changes was the gastric angulus in 73% (8/11) of NHPHi‐MNG cases and 67% (8/12) of NHPHi‐NG cases, compared to 98% (45/46) of HPi‐NG cases, which showed nodularity predominantly in the antrum (Figure 3B). These differences were significant (NHPHi‐MNG vs. HPi‐NG: *p* < 0.001; NHPHi‐NG vs. HPi‐NG: *p* < 0.001). The distance from the angulus to the proximal end of the nodular lesion was significantly longer in the NHPHi‐MNG (3.5 ± 1.0 cm) and NHPHi‐NG (3.1 ± 0.7 cm) groups than in the HPi‐NG group (1.7 ± 1.6 cm) (NHPHi‐MNG vs. HPi‐NG: *p* < 0.001; NHPHi‐NG vs. HPi‐NG: *p* = 0.007) (Figures 2G–I and 3C).

In terms of morphology (Figure 2J–O), the maximum nodule diameter was significantly larger in the NHPHi‐MNG group (3.3 ± 0.7 mm) than in the NHPHi‐NG (2.3 ± 0.5 mm) and HPi‐NG (2.1 ± 0.4 mm) groups (NHPHi‐MNG vs. NHPHi‐NG: *p* < 0.001; NHPHi‐MNG vs. HPi‐NG: *p* < 0.001) (Figure 3D). Regarding size heterogeneity, heterogeneous nodules were observed in 64% (7/11) of the NHPHi‐MNG group, but were absent in the NHPHi‐NG group (0/12) and found in only 9% (4/46) of the HPi‐NG group (Figure 3E). These differences were significant (NHPHi‐MNG vs. NHPHi‐NG: *p* = 0.004; NHPHi‐MNG vs. HPi‐NG: *p* < 0.001). Regarding nodule shape, the protruded type was significantly more frequent in the NHPHi‐MNG group (73%, 8/11) compared to 0% (0/12) in the NHPHi‐NG group and 13% (6/46) in the HPi‐NG group (NHPHi‐MNG vs. NHPHi‐NG: *p* = 0.001; NHPHi‐MNG vs. HPi‐NG: *p* < 0.001) (Figure 3F).

Interobserver agreement for continuous measurements ranged from good to excellent. The distance from the gastric angulus to the proximal end of the nodular lesion showed ICC(2,1) = 0.876 (95% CI 0.822–0.917), and the maximum nodule diameter showed ICC(2,1) = 0.741 (95% CI 0.644–0.821).

### 3.3 Comparison of Representative Histological Features of Nodular Lesions

Representative histological images are shown in Figure 2P–R. In the NHPHi‐MNG group, there was marked lymphocytic infiltration, and the presence of follicle‐like structures, which lacked polarity, showed irregular shapes because of fusion with adjacent structures and varied in size. In contrast, the NHPHi‐NG and HPi‐NG groups exhibited small, uniform lymphoid follicles with preserved polarity. Correlation between endoscopic and pathological measurements: the maximum endoscopic nodule diameter showed a significant positive correlation with the maximum lymphoid follicle diameter on H&E sections (Pearson *r* = 0.691, *p* < 0.001; *n* = 26) (Figure S1).

### 3.4 Diagnostic Performance of Endoscopic Features in Differentiating Nodular Lesions Among NHPHi‐MNG, NHPHi‐NG, and HPi‐NG

To differentiate among NHPHi‐MNG, NHPHi‐NG, and HPi‐NG, we evaluated the diagnostic performance (sensitivity, specificity, and accuracy) of various endoscopic features (Table 2). For continuous variables, including the distance from the angulus to the proximal lesion edge and the maximum nodule diameter, receiver operating characteristic (ROC) curve analyses were used to determine optimal cutoff values, which were then converted into categorical variables (Figure S2). The optimal cutoff values identified by ROC analysis for distinguishing NHPHi‐MNG from NHPHi‐NG were 5.0 cm for the distance from the angulus to the proximal end of the nodular lesion and 3.0 mm for the maximum nodule diameter. For comparisons between NHPHi‐NG and HPi‐NG, the cutoff values were 2.5 cm and 3.0 mm, respectively. The same values were applied for the comparison between NHPHi‐MNG and HPi‐NG groups. These thresholds were subsequently used as categorical variables in the respective analyses.

**TABLE 2:** Endoscopic differentiation among NHPHi‐MNG vs. NHPHi‐NG, NHPHi‐MNG vs. HPi‐NG, and NHPHi‐NG vs. HPi‐NG.

	Endoscopic features	Sensitivity	Specificity	Accuracy rate	Number of endoscopic predictive factors	Sensitivity	Specificity	Accuracy rate
Endoscopic features differentiating NHPHi‐MNG from NHPHi‐NG	Maximum diameter of nodules (≥ 3.0 mm)	0.909	0.667	0.783	1 or more	1.000	0.667	0.826
	Size heterogeneity of nodules (heterogeneous)	0.636	1.000	0.826	2 or more	0.818	1.000	0.913
	Shape of nodules (protruded)	1.000	0.800	0.870	3 or more	0.455	1.000	0.739
Endoscopic features differentiating NHPHi‐MNG from HPi‐NG	Sex (man)	0.727	0.761	0.754	1 or more	1.000	0.196	0.351
	Extent of nodularity (beyond the angulus)	1.000	0.435	0.544	2 or more	1.000	0.630	0.702
	Predominant site of nodularity (angulus)	0.727	0.978	0.930	3 or more	1.000	0.804	0.842
	Distance from the gastric angulus to the proximal end of nodular lesion (≥ 2.5 cm)	1.000	0.696	0.754	4 or more	0.909	0.935	0.930
	Maximum diameter of nodules (≥ 3.0 mm)	0.909	0.870	0.877	5 or more	0.818	0.957	0.930
	Size heterogeneity of nodules (heterogeneous)	0.636	0.913	0.860	6 or more	0.636	1.000	0.930
	Shape of nodules (protruded)	0.727	0.870	0.842	7 or more	0.364	1.000	0.877
Endoscopic features differentiating NHPHi‐NG from HPi‐NG	Extent of nodularity (beyond the angulus)	1.000	0.435	0.552	1 or more	1.000	0.413	0.534
	Predominant site of nodularity (angulus)	0.667	0.978	0.914	2 or more	1.000	0.717	0.776
	Distance from the gastric angulus to the proximal end of nodular lesion (≥ 2.5 cm)	1.000	0.696	0.759	3 or more	0.667	0.978	0.914

Abbreviations: HP, *Helicobacter pylori* ; HPi‐NG, HP‐induced nodular gastritis; NHPH, non‐*Helicobacter pylori Helicobacter*; NHPHi‐MNG, NHPH‐induced gastric mucosa‐associated lymphoid tissue lymphoma with a nodular gastritis‐like appearance; NHPHi‐NG, NHPH‐induced nodular gastritis.

In differentiating NHPHi‐MNG from NHPHi‐NG, three endoscopic features showed good discriminatory performance: a maximum nodule diameter ≥ 3.0 mm (sensitivity: 0.909, specificity: 0.667, accuracy: 0.783), heterogeneity in nodule size (sensitivity: 0.636, specificity: 1.000, accuracy: 0.826), and a protruded nodule type (sensitivity: 1.000, specificity: 0.800, accuracy: 0.870). When at least two of these features were present, diagnostic accuracy increased to 0.913, with a sensitivity of 0.818 and specificity of 1.000.

In distinguishing NHPHi‐MNG from HPi‐NG, several features demonstrated high diagnostic performance: male sex (sensitivity: 0.727, specificity: 0.761, accuracy: 0.754), extent of nodularity beyond the angulus (sensitivity: 1.000, specificity: 0.435, accuracy: 0.544), predominant site of nodularity at the angulus (sensitivity: 0.727, specificity: 0.978, accuracy: 0.930), proximal lesion boundary ≥ 2.5 cm from the angulus (sensitivity: 1.000, specificity: 0.696, accuracy: 0.754), maximum nodule diameter ≥ 3.0 mm (sensitivity: 0.909, specificity: 0.870, accuracy: 0.877), size heterogeneity (sensitivity: 0.636, specificity: 0.913, accuracy: 0.860), and protruded type (sensitivity: 0.727, specificity: 0.870, accuracy: 0.842). When ≥ 4 of these features were present, the diagnostic accuracy reached 0.930, with a sensitivity of 0.909 and specificity of 0.935.

In the comparison between NHPHi‐NG and HPi‐NG, three features were significant discriminators: nodularity extending beyond the angulus (sensitivity: 1.000, specificity: 0.435, accuracy: 0.552), predominant nodularity at the angulus (sensitivity: 0.667, specificity: 0.978, accuracy: 0.914), and distance from the angulus to the proximal lesion edge ≥ 2.5 cm (sensitivity: 1.000, specificity: 0.696, accuracy: 0.759). When all three features were present, the diagnostic accuracy increased to 0.914, with a sensitivity of 0.667 and specificity of 0.978.

### 3.5 Differential Algorithm to Distinguish Among HPi‐NG, NHPHi‐NG, and NHPHi‐MNG

**FIGURE 4:**
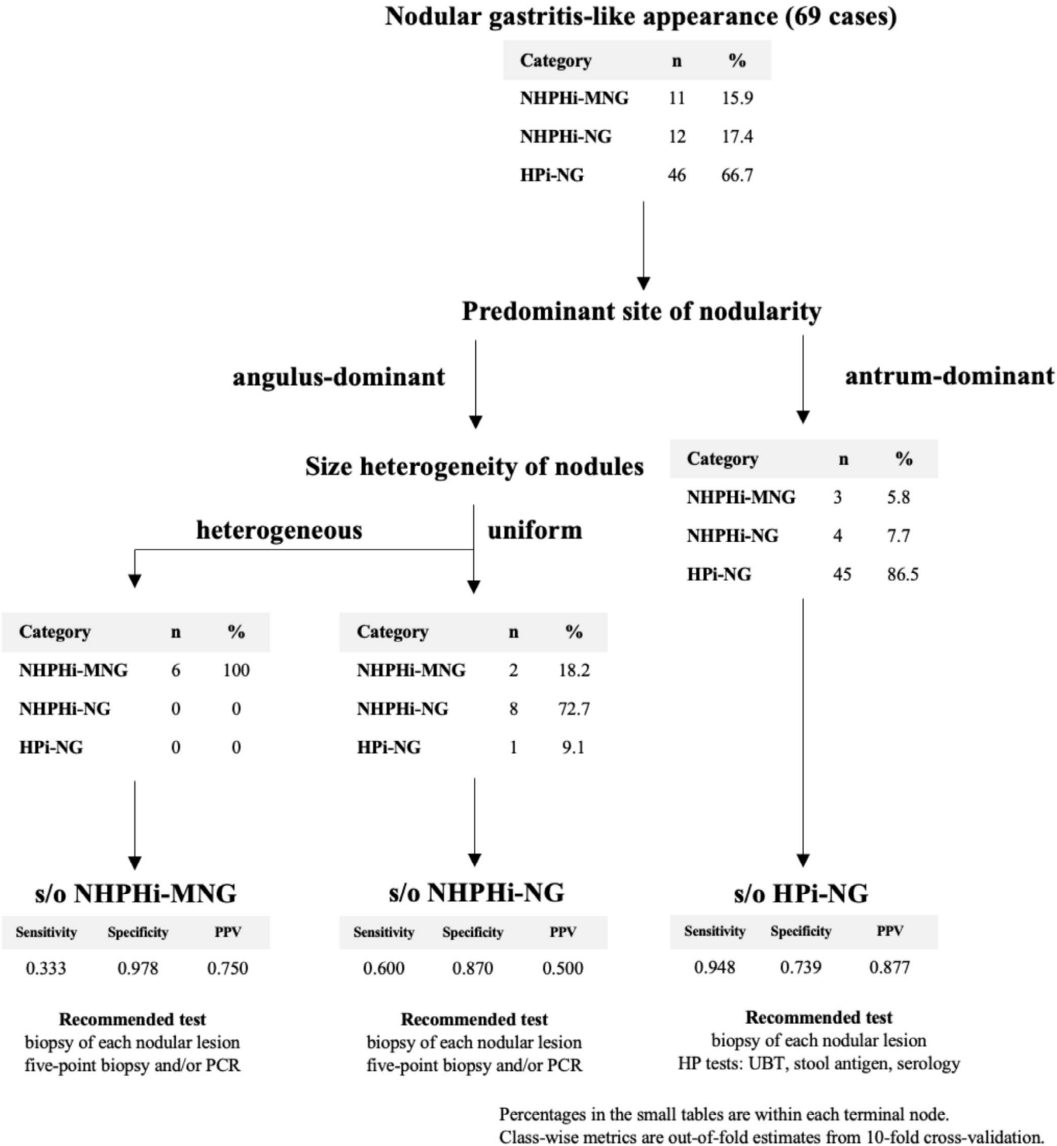
CART–based algorithm to differentiate HPi‐NG, NHPHi‐NG, and NHPHi‐MNG. The tree was trained on a stratified training set and pruned by the 1‐SE rule. The first split is the predominant site of nodularity (angulus‐dominant vs. antrum‐dominant), followed by size heterogeneity of nodules (heterogeneous vs. uniform). The small tables within each leaf show counts and within‐leaf percentages for the entire cohort (*n* = 69) for illustration; under each terminal node, class‐wise metrics (sensitivity, specificity, and PPV) are shown as out‐of‐fold estimates from stratified 10‐fold cross‐validation. Suggested next diagnostic tests are listed beneath each terminal node. CART, classification; HP, *Helicobacter pylori* ; HPi‐NG, HP‐induced nodular gastritis; NHPH, non‐*Helicobacter pylori Helicobacter*; NHPHi‐MNG, NHPH‐induced gastric mucosa‐associated lymphoid tissue lymphoma with a nodular gastritis‐like appearance; NHPHi‐NG, NHPH‐induced nodular gastritis; PPV, positive predictive value.

We analyzed 69 cases, split 80/20 into a stratified training set (*n* = 56) and an independent test set (*n* = 13). A pruned CART (rpart, R) generated a two‐step flow: first split by predominant nodular site (antrum vs. angulus) to infer organism group; among antrum‐predominant cases, size heterogeneity was then assessed for MALT involvement, yielding terminal nodes for NHPHi‐MNG, NHPHi‐NG, and HPi‐NG (Figure 4). Node counts/percentages display the full cohort (*n* = 69); performance was derived from cross‐validation and the independent test.

Performance was moderate (CV/independent test): overall accuracy 0.786/0.846 (95% CI 0.546–0.981 for the latter), Cohen's *κ* 0.552/0.606, and macro‐balanced accuracy 0.744/0.750; class‐wise sensitivity/specificity/PPV (CV → test) were NHPHi‐MNG 0.333/0.978/0.750 → 0.500/1.000/1.000, NHPHi‐NG 0.600/0.870/0.500 → 0.500/1.000/1.000, and Hpi‐NG 0.948/0.739/0.877 → 1.000/0.500/0.818.

## 4 Discussion

In this study, we compared three groups—NHPHi‐MNG, NHPHi‐NG, and HPi‐NG—to examine the morphological characteristics and distribution patterns of nodular endoscopic findings. In the NHPHi‐MNG group, nodules were large, protruded, and heterogeneous, densely distributed from the antrum to the lower corpus, with the most prominent nodules frequently observed in the angulus. In contrast, both the NHPHi‐NG and HPi‐NG groups exhibited small, flat, and uniform nodules. In NHPHi‐NG, these nodules extended from the antrum to the lower corpus, whereas in HPi‐NG, they were predominantly confined to the antrum (Figure 2A–O). These findings suggest that the distribution of nodular lesions is primarily influenced by the infecting *Helicobacter* species, whereas morphological features are more closely associated with neoplastic transformation. Notably, all NHPH‐positive cases in this study were identified as *H. suis* , raising the possibility that nodular endoscopic features associated with NHPH infection may be specific to this species.

Although the distribution of nodular lesions differed between the NHPHi‐MNG/NHPHi‐NG and HPi‐NG groups, this likely reflects species‐specific colonization preferences (Figure 3A–C) shaped by physiological adaptation to the gastric milieu. Because HP, which poorly tolerates strong acid, preferentially colonizes the antrum in early infection—often associated with nodular gastritis—guided by chemotaxis along pH gradients toward less acidic niches [46, 47, 48]. Its mucin binding peaks near neutral pH (6–7) and declines in acid [49, 50], making the low‐acid, mucus‐rich antrum favorable; with progressive antral atrophy, rising corpus pH can permit proximal spread [51]. By contrast, *H. suis* is acid‐adapted: it binds acidic mucins and persists at low pH [52], dies near neutral pH but grows around pH 5 [18], and therefore favors more acidic sites such as the angulus and adjacent corpus, where it may promote lymphoid follicle formation [53, 54]. In an additional exploratory analysis, we observed a significant positive monotonic association between the Kimura–Takemoto grade and the proximal extension of nodular lesions (Spearman's *ρ* = 0.608, *p* < 0.001; Figure S3), suggesting that as atrophy progresses, nodularity tends to extend beyond the angulus toward the corpus. Moreover, site‐specific *H. suis* PCR positivity showed a graded pattern—highest at the angulus, followed by the antrum, and lowest in the corpus (Figure S4). Taken together, these findings suggest a link between the spatial distribution of bacterial colonization and the endoscopic distribution of nodularity.

Regarding morphology, the nodules varied markedly depending on the presence or absence of neoplastic transformation (NHPHi‐MNG vs. NHPHi‐NG and HPi‐NG), consistent with our previous findings (Figure 3D–F) [7]. Histologically, nodular gastritis is characterized by intense mucosal inflammation with reactive lymphoid follicle hyperplasia in the lamina propria, which underlies its endoscopic appearance. In benign gastritis, these follicles are typically uniform in size and retain normal polarity. In contrast, MALT lymphoma involves neoplastic lymphoid proliferation forming irregular, enlarged, and often confluent follicle‐like structures (Figure 2P–R) [19]. These pathological features likely explain the uneven, enlarged, and protruded nodules observed endoscopically in MALT lymphoma cases. Consistent with these observations, the positive correlation between endoscopic maximum nodule diameter and maximum lymphoid follicle diameter on H&E sections provides quantitative pathological support for using maximum nodule diameter as the primary endpoint (Figure S1).

Furthermore, we assessed the diagnostic performance of endoscopic features in distinguishing among the three groups (Table 2). Differentiation between NHPHi‐MNG and NHPHi‐NG was achieved with 91.3% accuracy when at least two of the three morphological criteria—maximum diameter, size heterogeneity, and nodule shape—were present. Distinction between NHPHi‐MNG and HPi‐NG reached 93.0% accuracy when ≥ 4 of the following seven criteria were fulfilled: the three morphological features, three distributional characteristics (extent of nodularity, extension to the lower gastric body, and predominant nodular location), and patient sex. Differentiation between NHPHi‐NG and HPi‐NG achieved 91.4% accuracy when all three distributional indicators were met.

Compared with our previous report [7], the quantitative indices for NHPHi‐MNG showed minor divergence, whereas those for HPi‐NG remained largely consistent. This likely reflects cohort differences in case mix and lesion distribution (greater proximal extension in the prior cohort vs. a slightly higher share of larger nodules in the present cohort), as well as the inherently greater heterogeneity and smaller numbers of NHPHi‐MNG relative to the more uniform HPi‐NG. Accordingly, the distance cutoff shifted modestly, while the diameter cutoff was unchanged. Measurement procedures and equipment were broadly comparable across cohorts.

Finally, we constructed a bedside decision tree with two sequential steps—predominant site (angulus vs. antrum) followed by size heterogeneity—to classify NHPHi‐MNG, NHPHi‐NG, and HPi‐NG (Figure 4). Its performance was moderate, with reduced sensitivity for NHPHi‐MNG/NHPHi‐NG and reduced specificity for HPi‐NG; thus, the tree may guide test selection but should not replace clinical judgment. Given the limited sensitivity for NHPHi‐MNG, we recommend targeted biopsy of a representative nodule in all nodular cases. If NHPH is suspected, additional five‐point biopsies and/or PCR should be performed, whereas if HP is suspected, HP testing should be prioritized, with NHPH testing added when results are negative or indeterminate.

In the post‐eradication era, NHPH‐related disease is increasingly recognized [10, 11], yet routine diagnostics remain non‐standardized. This highlights the value of practical endoscopic cues such as those evaluated in the present study. Furthermore, in early‐stage lesions, histological evaluation may be inconclusive because of the presence of borderline changes, where distinguishing between reactive and neoplastic features is difficult. This distinction is crucial, as it directly affects decisions regarding further imaging and subsequent treatment planning. The principal clinical implication of this study is the enhanced ability to distinguish gastric MALT lymphoma—which frequently mimics nodular gastritis—from benign nodular gastritis. Moreover, inferring the infecting species (including differentiating NHPHi‐NG from HPi‐NG) is clinically meaningful. With increasing antimicrobial resistance, the primary success rate of HP eradication is variable and requires rigorous post‐treatment confirmation [55, 56, 57], whereas NHPH infections have been reported to respond relatively well to current eradication regimens [13, 58]. Thus, species inference may help refine testing and follow‐up strategies according to the expected treatment response. Nonetheless, eradication therapy remains the standard initial approach irrespective of species, and its impact on treatment selection is therefore limited.

We compared three entities with nodular endoscopic findings—NHPHi‐MNG, NHPHi‐NG, and HPi‐NG. Although HP is a major cause of gastric MALT lymphoma [59, 60, 61], most cases are superficial and nodular gastritis–like appearances are rare [36, 62]. Accordingly, we enrolled only lesions with a nodular appearance and classified cases by histology and *Helicobacter* testing. During the study period, no HP‐induced MALT lymphomas with this appearance were identified. All PCR‐positive organisms in nodular lesions were *H. suis* , consistent with our previous series (all nodular NHPH cases due to *H. suis* ) [34] and with reports that, apart from HP, nodularity‐associated MALT lymphoma is most often caused by *H. suis* [4, 5, 18, 33, 63, 64]. In another series from our group, four of six cases were PCR‐positive for *H. suis*, and two showed NHPH histology without species identification; thus, NHPH species other than *H. suis* cannot be excluded [4, 16]. Overall, nodular formation among NHPH appears most closely linked to *H. suis* , but not all *H. suis* infections are nodular, implying additional factors beyond species. Going forward, as nodular cases caused by NHPH species other than *H. suis* accumulate, their endoscopic phenotypes will be a highly compelling focus of investigation. Our findings further suggest that the spatial distribution of nodularity may reflect species‐specific adaptation to gastric acidity and preferred intragastric niches. Accordingly, distributional differences may emerge for other NHPH species; however, this remains hypothetical, and data on their pH tolerance, mucin binding or chemotaxis, and intragastric localization are currently limited.

This single‐center retrospective cohort had a limited sample size, particularly for NHPH‐associated MALT lymphoma with nodularity. PCR was performed on nucleic acids extracted from routine‐care biopsies, restricting retrospective accrual. Thus, generalizability is limited, and multicenter validation is needed. Because of the retrospective design, endoscope models (Olympus, Tokyo, Japan: GIF‐H260Z, GIF‐HQ290, GIF‐H290Z, GIF‐EZ1500, and GIF‐XZ1200), zoom settings (optical/digital), observation distance, field of view, and insufflation (CO_2_ or room air) were not standardized, leading to variability in gastric distention. These factors may have influenced apparent nodule size and measured distances; mm/pixel calibration was not feasible, and some measurement errors may remain. Furthermore, prior studies indicate that nodular gastritis in younger patients is associated with relatively mild corpus inflammation and tends to regress as atrophy progresses with aging and persistent HP infection, whereas cases persisting into older age show higher corpus inflammatory infiltration and HP density despite mild atrophy [39, 65]. These observations suggest that age and the degree of atrophy may influence nodular endoscopic features. Our cohort was predominantly composed of younger to middle‐aged patients with mild atrophy; thus, the HPi‐NG nodular findings identified here are likely to reflect this specific age/atrophy spectrum. Accordingly, applicability to older patients or markedly atrophic stomachs should be interpreted with caution, and external validation in age‐stratified cohorts is recommended.

In this study, we focused on three distinct conditions characterized by nodular findings on endoscopy—NHPHi‐MNG, NHPHi‐NG, and HPi‐NG—and conducted a detailed comparative analysis of nodular morphology and distribution. Our results indicate that morphological features, such as nodule size, heterogeneity, and elevation, are closely associated with the presence or absence of neoplastic transformation. In contrast, differences in nodular distribution appear to be primarily influenced by the infecting *Helicobacter* species. By integrating these endoscopic features, high diagnostic accuracy can be achieved based on endoscopic observation alone. In cases where definitive diagnosis is challenging, these visual findings may complement pathological assessment and adjunct testing, thereby enhancing diagnostic precision and informing appropriate clinical management. Overall, our findings support the development of a novel diagnostic algorithm for nodular gastritis in the post‐eradication era.

## Author Contributions

Yuki Kitadai and Hidehiko Takigawa conceived the study and designed the experiments. Yuki Kitadai, Hidehiko Takigawa, Akinori Nagao, Daisuke Shimizu, Misa Ariyoshi, Takeshi Takasago, Ken Yamashita, and Yuichi Hiyama analyzed and interpreted the data. Yuki Kitadai, Hidehiko Takigawa, Akiyoshi Tsuboi, Hidenori Tanaka, Yoshihiro Kishida, and Yuji Urabe contributed to patient enrollment. Akira Ishikawa conducted the pathological evaluations. Toshio Kuwai and Shiro Oka reviewed and approved the final manuscript.

## Ethics Statement

The study was approved by the Institutional Review Board of Hiroshima University Hospital (E‐298) and conducted in accordance with the Declaration of Helsinki. Given the retrospective use of anonymized data, the requirement for informed consent was waived, with an opt‐out option. No animal experiments were performed, and the study was not registered as a clinical trial.

## Conflicts of Interest

The authors declare no conflicts of interest.

## Supporting information


**Figure S1:** Correlation between endoscopic nodule size and histological follicle size.
**Figure S2:** ROC analyses of key endoscopic metrics for pairwise discrimination.
**Figure S3:** Association between mucosal atrophy and proximal extension of nodularity in HPi‐NG.
**Figure S4:** Site‐specific *H. suis* PCR positivity by gastric biopsy site.

## Data Availability

The data that support the findings of this study are available from the corresponding author upon reasonable request.
