# Supplementary material for: A Three‐Way Comparison of Nodular Lesions in H. pylori‐Induced Gastritis, Non‐Helicobacter pylori Helicobacter (NHPH)‐Induced Gastritis, and NHPH‐Induced MALT Lymphoma Reveals Their Distinct Endoscopic Predictors: Distribution for Bacterial Etiology and Morphology for Malignancy
Source: Helicobacter. 2025 Oct 14;30(5):e70079. doi: 10.1111/hel.70079 (PMC12521800; doi:10.1111/hel.70079)
Supplement: Supplementary file 1 — Figure S1: Correlation between endoscopic nodule size and histological follicle size. Figure S2: ROC analyses of key endoscopic metrics for pairwise discrimination. Figure S3: Association between mucosal atrophy and proximal extension of nodularity in HPi‐NG. Figure S4: Site‐specific H. suis PCR positivity by gastric biopsy site. [file HEL-30-e70079-s001.docx]

**
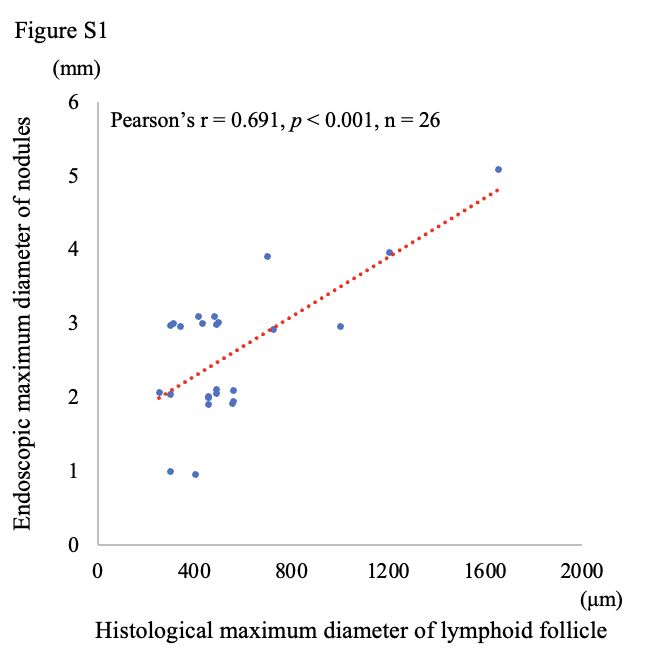
**

**Figure S1. Correlation between endoscopic nodule size and histological follicle size.**

Scatter plot of the endoscopic maximum diameter of nodules (mm) vs. the histological maximum diameter of lymphoid follicles on H&E sections (µm) in biopsy-proven follicle–positive cases (n=26). The red dotted line shows the least-squares fit (Pearson’s r = 0.691; p < 0.001). Points were slightly jittered to minimize overlap; all statistics and regression used the original (non-jittered) data.

**
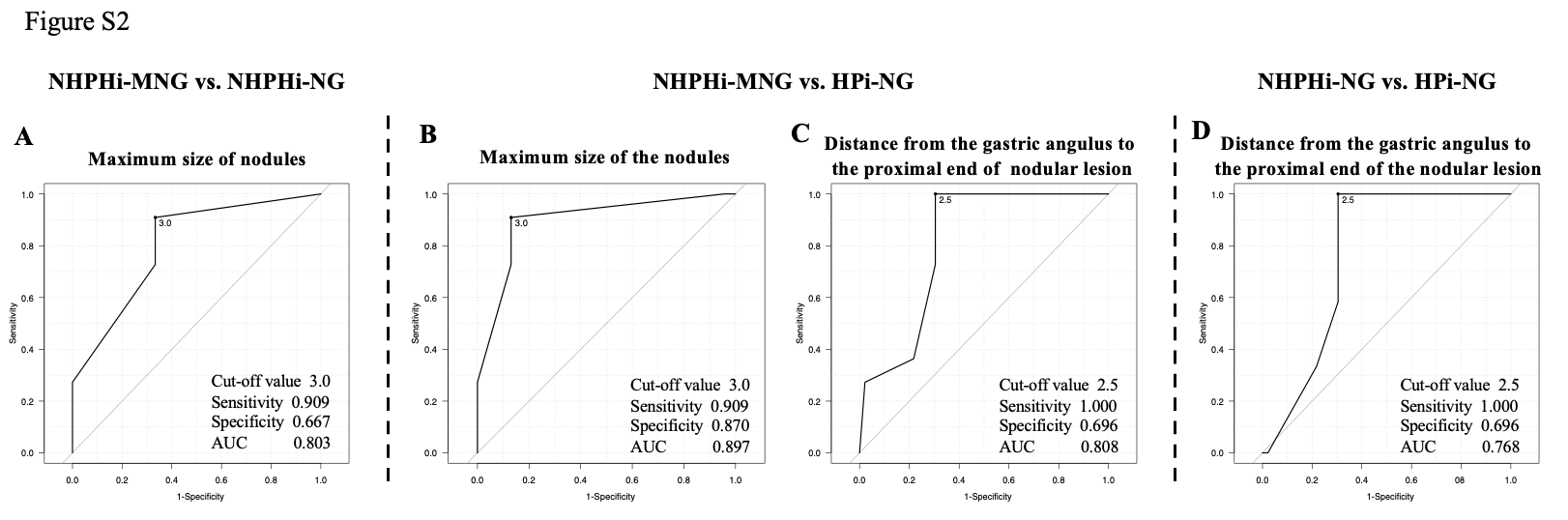
Figure S2. ROC analyses of key endoscopic metrics for pairwise discrimination.**

A: NHPHi-MNG vs. NHPHi-NG: maximum nodule diameter. Optimal cutoff 3.0 mm (sensitivity 0.909, specificity 0.667, AUC 0.803). B: NHPHi-MNG vs. HPi-NG: maximum nodule diameter. Cutoff 3.0 mm (sensitivity 0.909, specificity 0.870, AUC 0.897). C: NHPHi-MNG vs. HPi-NG: distance from the gastric angulus to the proximal edge of the nodular lesion. Cutoff 2.5 cm (sensitivity 1.000, specificity 0.696, AUC 0.808). D: NHPHi-NG vs. HPi-NG: same distance metric. Cutoff 2.5 cm (sensitivity 1.000, specificity 0.696, AUC 0.768). Only features with significant univariable discrimination (p < 0.05) are shown.

NHPH, non-*Helicobacter pylori Helicobacter*; HP, *Helicobacter pylori*; NHPHi-MNG, NHPH-induced gastric mucosa-associated lymphoid tissue lymphoma with a nodular gastritis-like appearance; NHPHi-NG, NHPH-induced nodular gastritis; HPi-NG, HP-induced nodular gastritis; ROC, receiver operating characteristic.

**
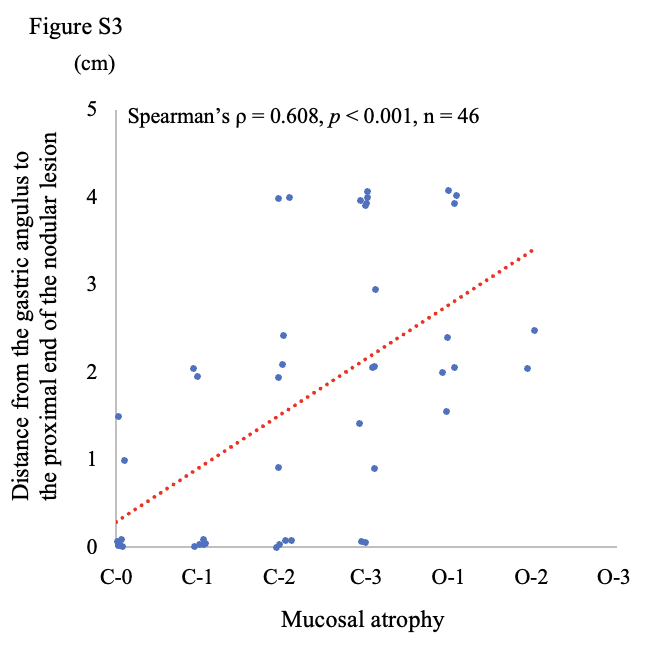
**

**Figure S3. Association between mucosal atrophy and proximal extension of nodularity in HPi-NG.**

Scatter plot showing the distance from the gastric angulus to the proximal end of nodular lesion (cm) across Kimura–Takemoto atrophy grades (C-0 to O-3) in HPi-NG cases (n=46). The red dotted line indicates the monotonic trend (Spearman’s ρ = 0.608; p < 0.001). Points were slightly jittered to reduce overlap; all statistics were calculated using the original (non-jittered) data.

C / O, closed-type / open-type.

**
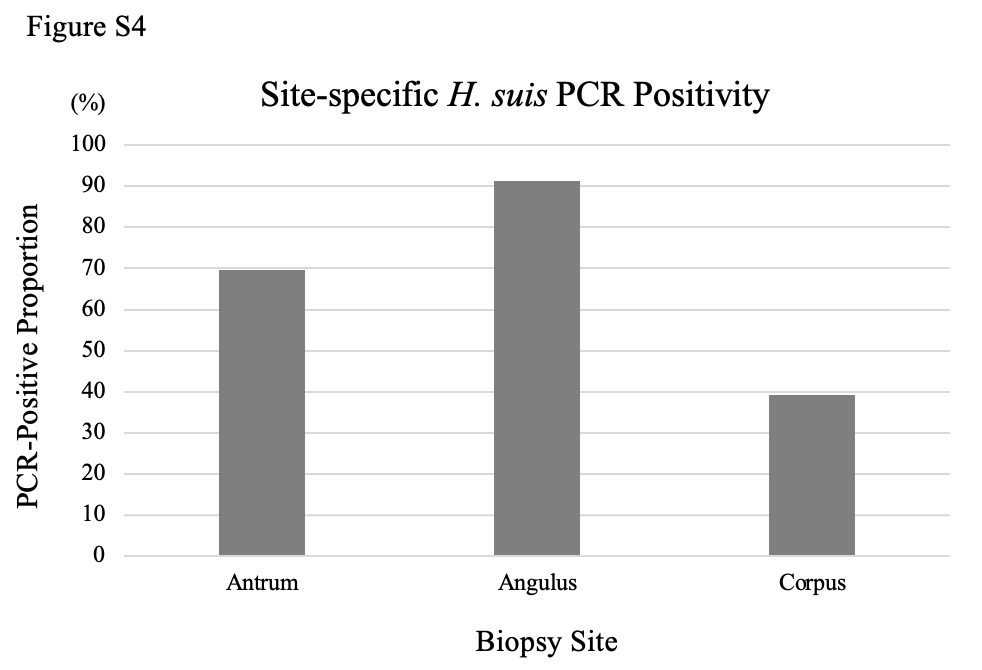
Figure S4. Site-specific *H. suis* PCR positivity by gastric biopsy site.**

Bar chart showing the proportion of PCR-positive specimens at each site (antrum, angulus, and corpus). The percentages were calculated as the number of *H. suis* PCR–positive biopsies divided by the number tested at each site. Positivity was highest at the angulus, intermediate in the antrum, and lowest in the corpus.

*H. suis*, *Helicobacter suis*; PCR, polymerase chain reaction.
